# CFD Investigation of the Effect of Condensation Chamber Geometry on Nanoparticle Transport in Magnetron Sputtering

**DOI:** 10.3390/nano16100599

**Published:** 2026-05-13

**Authors:** Lin Gao, Liye Zhao, Yue Dong

**Affiliations:** 1 School of Instrument Science and Engineering, Southeast University, Nanjing 210096, China; 220233690@seu.edu.cn; 2 Key Laboratory of Micro-Inertial Instruments and Advanced Navigation Technology, Ministry of Education, Nanjing 210096, China; 3 Nanjing Institute of Atomic-Scale Manufacturing, Nanjing 211800, China; dongyue@atomnju.ac.cn

**Keywords:** magnetron sputtering, CFD, axisymmetric flow, particle trajectory

## Abstract

In magnetron sputtering-based gas aggregation sources, nanoparticle formation and yield are strongly influenced by the flow-regulated transport and residence time of particles within the condensation chamber. However, the effect of internal geometric parameters on flow structure and nanoparticle growth is not well understood. In this study, computational fluid dynamics (CFD) coupled with a discrete phase model (DPM) is employed to investigate how magnetron radius affects flow characteristics, particle transport, and their implications for nanoparticle formation. The results show that increasing the magnetron radius significantly enhances axial flow uniformity and suppresses vortex structures near the inlet. This shift from radial diffusion-dominated to primarily axial transport effectively reduces particle trapping and wall deposition. Furthermore, the regulation of flow structure modifies particle residence time distributions, which is considered a key factor associated with nanoparticle growth potential and size evolution in gas-phase synthesis. Larger magnetron radii promote more stable transport pathways and improve particle transmission efficiency, thereby improving particle transmission efficiency and providing more favorable conditions for nanoparticle formation. These findings indicate that geometric optimization can simultaneously enhance transport efficiency and influence the conditions potentially favorable for particle growth, providing valuable guidance for the design of high-yield nanoparticle synthesis systems. Overall, this work provides insight into how flow field characteristics influence nanoparticle transport and potential growth behavior, offering a foundation for optimizing magnetron sputtering-based nanoparticle synthesis.

## 1. Introduction

In recent years, nanoparticle-based coatings and functional nanomaterials have attracted increasing attention due to their size-dependent electronic and structural properties [1], which are critical for applications in catalysis [2], coatings, biomaterials, nanoscale etching and smoothing processes [3].

Currently, common methods for cluster preparation include laser evaporation, sputtering, chemical vapor deposition (CVD), vapor condensation, and solution methods. Chemical methods typically offer higher yields, while physical methods, although having relatively lower yields, allow for more precise control over cluster size [4], composition [5], and structure [6].

Magnetron sputtering is a widely used physical vapor deposition (PVD) technique. In a high-vacuum environment, a magnetic field constrains electron motion to increase plasma density. Gas ions (typically argon ions) between the electrodes generate high-energy electrons that bombard the target surface, thereby sputtering atoms or molecules from the target. These sputtered atoms form nanoparticles that are subsequently transported in the gas phase and ultimately deposited onto the substrate surface to form a thin film [7]. Compared to other physical deposition methods, magnetron sputtering offers higher throughput [8]. However, magnetron sputtering currently faces throughput limitations that hinder large-scale nanoparticle production, as the deposition rates often fall short of industrial expectations.

When preparing silver nanoparticles using the magnetron sputtering method, high-energy ions typically form in the vicinity of the target and are transported to the outlet of the condensation chamber by the carrier gas. During transport, the particles are subject to various forces and collisions [9]. Some sputtered atoms may redeposit onto the target surface [10]; if they collide with the chamber wall while carried by the gas, they will be captured by the wall, resulting in loss; if they enter recirculation or vortex regions with the gas flow, they may remain trapped inside the chamber for extended periods. Therefore, the transport efficiency of nanoparticle to the subsequent mass selector is limited by vortices and low-temperature wall deposition in the carrier gas flow field, and depends on the fluid geometry within the condensation chamber. Furthermore, a longer residence time of particles within the chamber typically promotes nanoparticle growth, leading to larger particle sizes, whereas insufficient residence time limits nucleation and growth. Therefore, the flow field not only determines particle transport efficiency but also indirectly controls the size distribution and yield of nanoparticles.

S. Batkova used a gas-phase condensation method to prepare Cu nanoparticles and found that the pressure-to-flow ratio remains constant for a given pore size but varies with changes in pore size [11]. This ratio determines the drift velocity and residence time of the nanoparticles, and the nanoparticle size increases significantly as the pore size decreases.

Sanzone investigated the effects of chamber geometry and inlet location on particle flux and concluded that, compared to cylindrical chambers, a conical design with a smaller apex angle is most effective. By reducing the chamber diameter and the apex angle of the conical chamber, the gas velocity distribution was optimized, thereby increasing the gas velocity near the walls. Placing the gas inlet inside the magnetron source, rather than at the rear of the condensation chamber, helps reduce the redeposition of sputtered atoms on the magnetron target.

Zhang used OpenFOAM to study the effect of nozzle geometry on nanoparticles and concluded that nozzles with convergent cross-sections exhibit stronger aerodynamic focusing effects, and that the presence of Brownian diffusion does not eliminate the aerodynamic focusing effect of the nozzle [12].

While existing research has primarily focused on altering the aperture, geometry, and spatial arrangement of the condensation chamber outlet, as well as the influence of the chamber‘s overall geometry on convection field characteristics, the influence of the internal magnetron remains under-researched. As larger targets become necessary for scaling yield, the geometric parameters of the cylindrical magnetron located within the chamber may significantly influence the gas flow structure and particle transport efficiency within the chamber.

Although extensive research has been conducted on chamber geometry and outlet structures, the influence of the magnetron geometry within the condensation chamber on flow patterns, particle residence time, and nanoparticle throughput remains largely unexplored. In particular, as larger magnetron targets are adopted to increase production throughput, understanding how geometric scales affect nanoparticle formation becomes increasingly important.

In this work, we systematically investigated the influence of the magnetron radius on flow characteristics and particle transport behavior, with a particular focus on its implications for nanoparticle yield, growth potential, and overall production.

## 2. Mathematical Model

**Figure 1:**
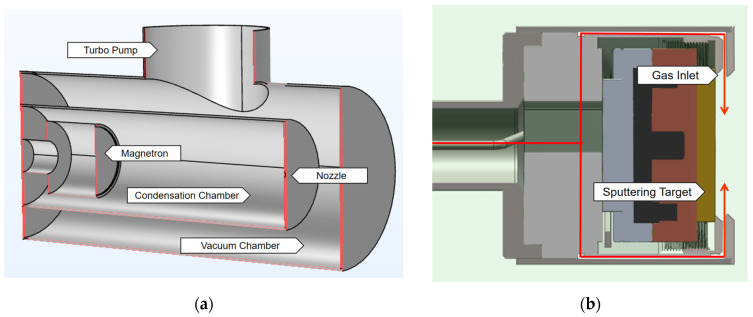
(**a**) Schematic diagram of a magnetron sputtering system (cross-section view); (**b**) 2D sketch of the magnetron. The gas is provided from within the magnetron.

The magnetron sputtering module of the nanoparticle synthesis apparatus incorporates a vacuum chamber, a condensation chamber, and a magnetron source. The vacuum system, integrated with mechanical pumps, molecular pumps, and associated piping, maintains the requisite high-vacuum environment for the sputtering process (Figure 1).

**Figure 2:**
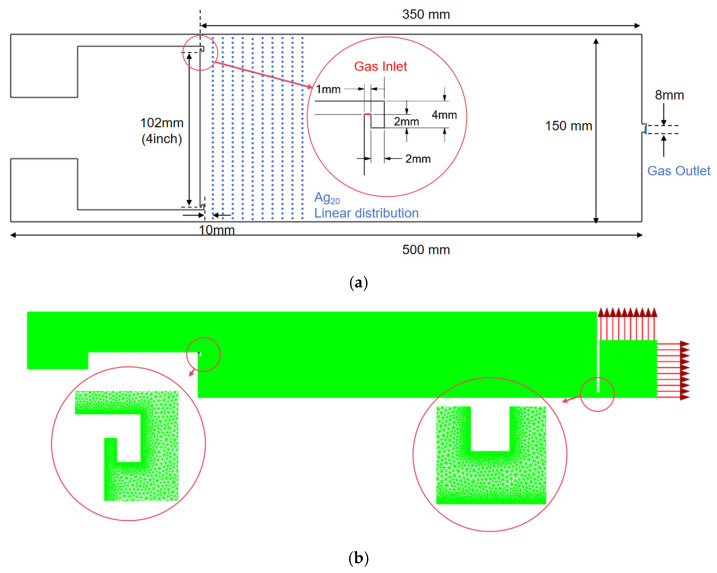
(**a**) 2D sketch of the condensation chamber. (**b**) Computational mesh.

The condensation chamber inside the vacuum chamber is the primary region where sputtered atoms combine and grow into nanoparticles. The magnetron is positioned at the base of the condensation chamber, while the target is fixed to its upper surface. A circular slit surrounding the target functions as the argon inlet. The cylindrical condensation chamber features a length of 500 mm, a radius of 75 mm, and an outlet radius of 4 mm. The magnetron measures 100 mm in length and includes a 50 mm retractable magnetron rod. Specifically, the target is secured to the magnetron head, with its active surface positioned 350 mm from the chamber outlet. To accommodate various scales, the support radii are configured at 41 mm, 54 mm, and 66.5 mm, corresponding to 3-inch, 4-inch, and 5-inch targets, respectively (Figure 2).

### 2.1. Control Equations

In this study, the flow field was simulated using the CFD simulation software Ansys Fluent 2024. The gas flow within the condensation chamber is treated as steady-state compressible flow, satisfying the equations of conservation of mass, momentum, and energy.

Given the geometric symmetry of the condensation chamber, the governing equations are solved in a 2D axisymmetric coordinate system, where the velocity field $\vec{v}=\left(v_{r},v_{z}\right)$ is a vector with radial and axial components.

The gas flow is governed by three conservation equations. The continuity equation is:

(1) $$\frac{\partial \rho }{\partial t}+\nabla \cdot (\rho \vec{v})=0,$$

The momentum conservation equation (compressible Navier–Stokes equation) is:

(2) $$\frac{\partial (\rho \vec{v})}{\partial t}+\nabla \cdot (\rho \vec{v}\vec{v})=-\nabla p+\nabla \cdot \overset{=}{\tau }+\rho \vec{g}+\vec{F},$$

(3) $$\overset{=}{\tau }=\mu \left[(\nabla \vec{v}+\nabla {\vec{v}}^{T})-\frac{2}{3}\nabla \cdot \vec{v}\cdot I\right],$$

where $\vec{F}$ is the external body force source term, set to zero in this study as gravitational and electromagnetic effects are neglected. *μ* denotes the dynamic viscosity of argon.

The energy conservation equation is:

(4) $$\frac{\partial (\rho E)}{\partial t}+\nabla \cdot \left[\vec{v}\left(\rho E+p\right)\right]=\nabla \cdot \left(k_{eff}\nabla T\right)+S_{h},$$

To determine whether the continuity assumption holds, the Knudsen number (*Kn*) is introduced:

(5) $$Kn=\frac{\lambda }{L}$$

where *λ* is the mean free path of gas molecules, and *L* is the characteristic length scale.

Under the operating conditions of this study, *p* = 60 Pa is the chamber operating pressure, and T = 77 K is the cold wall temperature, as a lower gas temperature generally speeds up the nanoparticle growth process [13]. Calculations based on the theory of gas molecular motion yield λ ≈ 3.4 × 10^−5^ m, thereby confirming that the argon flow operates within the continuum regime [14]. To obtain a conservative upper-bound estimate of the Knudsen number, the outlet radius L=4 mm is adopted as the characteristic length, as it represents the smallest geometric feature in the computational domain. The Knudsen number is therefore: Kn ≈ 8.5 × 10^−3^. Since Kn<0.01 even at this most restrictive location, the flow throughout the entire domain satisfies the continuum criterion. Consequently, the application of Navier–Stokes equations under the continuum assumption is justified.

Furthermore, the Reynolds number (Re) is introduced to characterize the flow state:

(6) Re=ρvLη

*ρ* is the fluid density, *v* is the characteristic velocity of the fluid, *L* is the characteristic length of the flow, and *η* is the dynamic viscosity of the fluid.

The relationship between the dynamic viscosity of argon and temperature can be approximated using the Sutherland equation:

(7) $$\eta =\eta_{0}{\left(\frac{T}{T_{0}}\right)}^{\frac{3}{2}}\frac{T_{0}+S}{T+S}$$

The calculation results indicate that Re ≈ 10^4^, which falls within the transition region between laminar and turbulent flow. Therefore, this study adopts the realizable k–ε turbulence model to simulate the flow field, which ensures a compromise between computational stability and predictive accuracy.

### 2.2. Particle Model

Given the nanometer scale of the nanoparticles, the influences of gravity and inter-particle collisions are deemed negligible. Particle dynamics are governed predominantly by gas-phase viscous drag and Brownian motion.

To evaluate the particles’ responsiveness to changes in the flow field, the Stokes number (*St*) is introduced, which indicates whether particles can move with the flow field [15]:

(8) $$St=\frac{\tau_{p}}{\tau_{f}}$$

where $\tau_{p}$ is the particle response time, and $\tau_{f}$ is the characteristic time of the flow field. Computational results indicate that *St* ≪ 1, indicating that particles exhibit a rapid response to local velocity fluctuations.

To assess the influence of non-continuum drag on particle transport, and the Cunningham-corrected Stokes number was evaluated. With $C_{c}\approx 1.16\times 10^{5}$, yielding a corrected Stokes number $St_{corr}\approx 1.7\times 10^{-5}$. This value remains far below unity, consistent with the uncorrected estimate of $St_{Stokes}\approx 1.5\times 10^{-10}$. Both estimates confirm St≪1, meaning that ${\mathrm{Ag}}_{20}$ closely follows local fluid streamlines under either drag assumption. The relative transmission efficiency trends between magnetron configurations are therefore independent of this correction.

As the particles exert negligible feedback on the gas phase, a one-way coupled Lagrangian particle tracking method is employed.

Particle trajectory tracking is performed using the DPM in Fluent. In magnetron sputtering-based gas aggregation sources, nanoparticles nucleate near the target surface as sputtered atoms undergo collisions and coalescence in the carrier gas. In this study, ${\mathrm{Ag}}_{20}$—a cluster of 20 silver atoms with an equivalent spherical diameter of approximately 1 nm and a mass of 3940 amu—is adopted as a representative model particle. This size corresponds to nascent nanoclusters at the onset of the nucleation process, lying at the boundary between atomic clusters and nanoparticles as conventionally defined. Once a particle comes into contact with the wall, it is considered to be completely captured and removed from the computational domain.

After solving for the steady-state solution of the carrier fluid domain, particles are uniformly released from a cross-section 10 to 90 mm in front of the target, perpendicular to the axis, with an initial velocity assumed to be consistent with the local gas velocity [16].

This research emphasizes the impact of flow field pattern on particle transport. Accordingly, the simulation is simplified by excluding the complex nanoparticle nucleation and growth kinetics, and does not pre-determine the initial formation coordinates. Although nanoparticle nucleation and growth kinetics are not explicitly modeled in this study, particle residence time is used as an indirect indicator of growth potential, as longer residence times generally favor nanoparticle coalescence and size evolution in gas-phase synthesis.

### 2.3. Boundary Conditions and Computational Methods

A steady-state solution method based on a density-based solver was employed to numerically solve the equations of conservation of mass, momentum, and energy. Turbulence effects are described using the realizable k–ε model [17].

The model includes the energy equation. The magnetron sputtering process requires the pressure within the condensation chamber to be maintained within a narrow range (10–100 Pa). The initial pressure in the condensation chamber is set to 60 Pa. Only argon, the working gas, is considered in the flow field, and argon is treated as an ideal gas.

The inlet boundary is set as a mass flow inlet with a mass flow rate of 4.5 × 10^−6^ kg/s, corresponding to a volumetric flow rate of 150 sccm. To investigate flow effects, the flow rate is further increased to 300 sccm and 450 sccm for comparative analysis. The outlet boundary is set as a pressure outlet with a gauge pressure of 0.1 Pa. All chamber walls are defined as stationary, non-slip surfaces. In the DPM, all walls are set to Trap mode, including the target surface, as some nanoparticles will be deposited back to the target surface [18].

Convergence was assessed by monitoring the scaled residuals of all governing equations. The solution was considered converged when all residuals fell below 10^−3^ for energy, continuity, momentum, and turbulence quantities. Additionally, the mass flow rate at the outlet was monitored as a global convergence indicator and confirmed to be stable to within 0.1% over the final 200 iterations.

It is also noted that low-Mach-number preconditioning, available within the density-based solver framework, was not employed in the present study. Activating this option in future simulations involving similar mixed-Mach flow configurations may further improve convergence efficiency, particularly in the low-velocity cavity regions where Ma < 0.1.

### 2.4. Mesh-Independence Verification

An increase in the magnetron radius constricts the annular gap between the magnetron and the chamber wall. To ensure that the calculation results are not affected by the mesh, a mesh-independence verification is required. While maintaining consistency in the geometric model, boundary conditions, and solution parameters, three sets of computational meshes with different numbers of elements were generated using Pointwise. The number of mesh elements was approximately 0.5 × 10^6^, 0.8 × 10^6^, and 1.3 × 10^6^, respectively. Since this study primarily focuses on the flow field structure and particle transport behavior within the cavity, three indicators were selected to evaluate mesh independence: (1) the area-weighted average velocity at the outlet, representing bulk flow behavior; (2) the area of the region with vorticity magnitude exceeding 40 s^−1^ within the main deposition zone (target surface to 100 mm from the target), serving as a flow-structure indicator sensitive to vortex topology; and (3) the particle transmission rate at 10 mm from the target surface, serving as a particle-transport indicator directly relevant to deposition characteristics.

Mesh quality parameters were maintained within acceptable ranges, with a maximum skewness of less than 0.85 and an orthogonality of greater than 0.25 (Table 1).

It can be observed that the variation between Mesh 1 and Mesh 2 is less than 2%. Therefore, Mesh 2, with approximately 0.8 × 10^6^ elements, was selected for subsequent simulations to balance computational accuracy and efficiency (Table 2).

## 3. Results and Discussion

### 3.1. Influence of Magnetron Radii on Flow Field Characteristics

**Figure 3:**
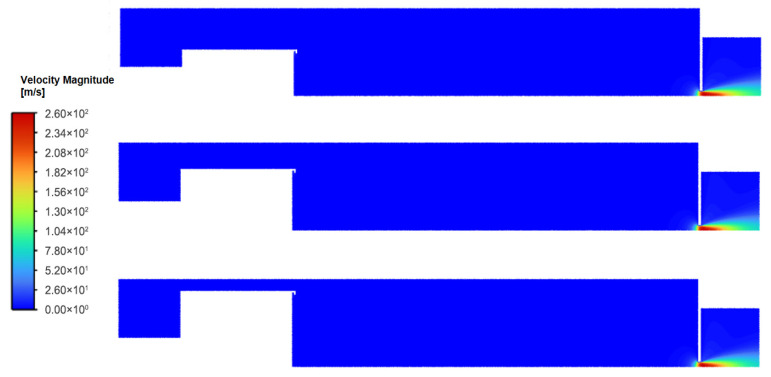
Velocity distribution in the flow field for different magnetron radii at 150 sccm.

**Figure 4:**
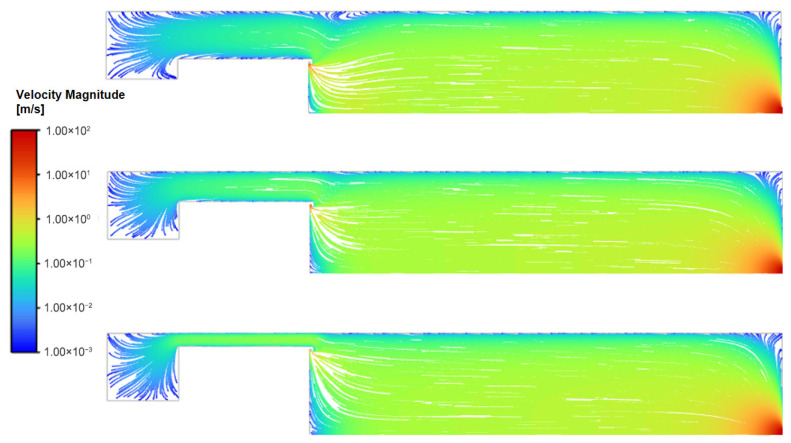
Streamline diagram for different magnetron radii at 150 sccm.

For the range of magnetron radius, the overall flow field structure within the condensation chamber remains highly consistent (Figure 3 and Figure 4). The working gas, argon, enters the chamber through the annular inlet surrounding the target, resulting in localized high-velocity flow in the inlet region, with velocities ranging from approximately 1–10 m/s. Since the total mass flow rate remains constant, as the magnetron radius increases, the inlet area expands, thereby leading to a marginal reduction in the effective inlet velocity.

The gas velocity in the main body of the chamber rapidly decreases to approximately 0.1 m/s, with the flow dominated by axial transport. Approaching the outlet, the significant pressure gradient between the chamber interior and the nozzle induces rapid gas acceleration [19], reaching a peak velocity of roughly 200 m/s.

Overall, the flow field exhibits typical axisymmetric distribution characteristics. There are no obvious large-scale recirculation structures in the main flow region; only small-scale vortices exist locally at geometric discontinuities (such as the junction between the chamber bottom and side walls). Particles carried by a fluid tend to concentrate in the low-vorticity regions at the edges of vortices, effectively acting as hydrodynamic guide structures [20].

**Figure 5:**
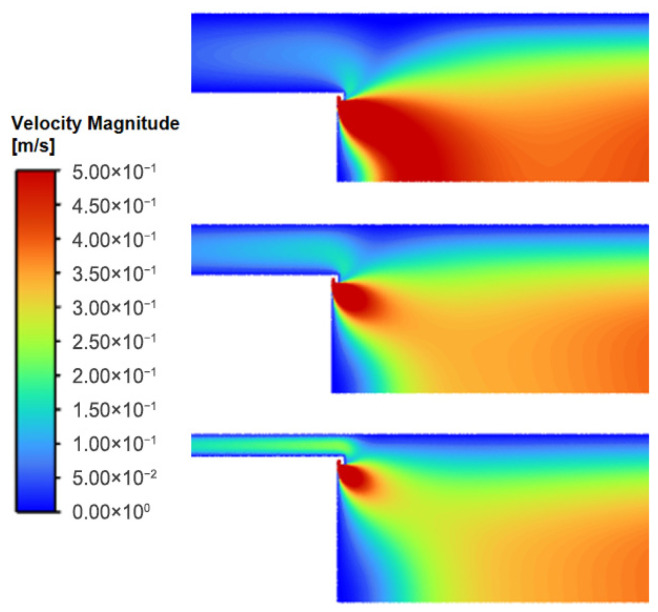
Velocity in the annular gap between the magnetron and the chamber wall.

An increase in the magnetron radius constricts the annular gap between the magnetron and the chamber wall, elevating the local average velocity to 0.08 m/s, 0.10 m/s, and 0.12 m/s, respectively (Figure 5). As the magnetron radius increases, the annular gap narrows and axial flow velocity within the gap increases. This constriction redirects the streamlines near the chamber wall toward the axial direction, reducing streamline curvature and suppressing flow separation. Consequently, the flow field transitions from a pattern characterized by strong radial diffusion to one dominated by axial transport. This global structural shift has direct implications for particle transport efficiency, as discussed in Section 3.2.

**Figure 6:**
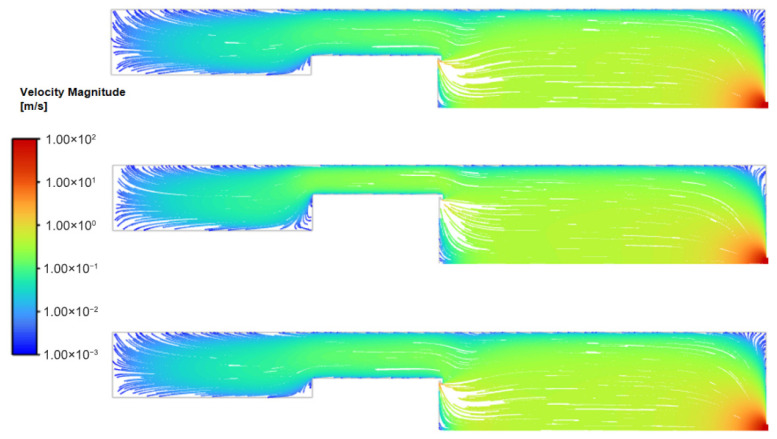
Streamline diagram for different magnetron radii at 150 sccm (target–nozzle distance 250 mm).

When the magnetron rod was extended, with the target surface 250 mm from the outlet, the fluid velocity field is shown in Figure 6. The overall trend is similar to that at 350 mm; the particle transmission rate near the target surface has increased, and the higher the magnetron radius, the higher the particle transmission rate.

Overall, changing the cylinder diameter did not significantly alter the overall flow field topology within the chamber; however, by adjusting the local velocity distribution—particularly the flow characteristics in the annular gap region and near the outlet—differences in particle transport behavior were observed.

### 3.2. Influence of Magnetron Radii on Particle Throughput at Different Mass Flows

**Figure 7:**
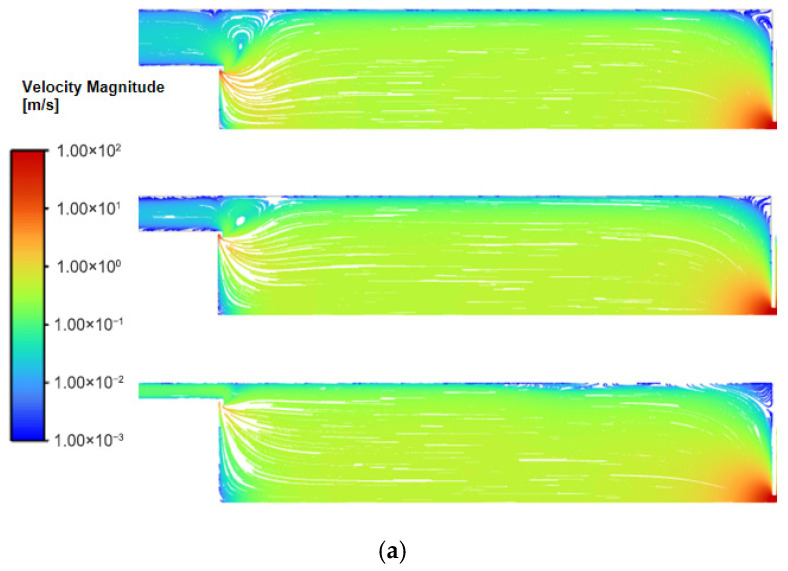

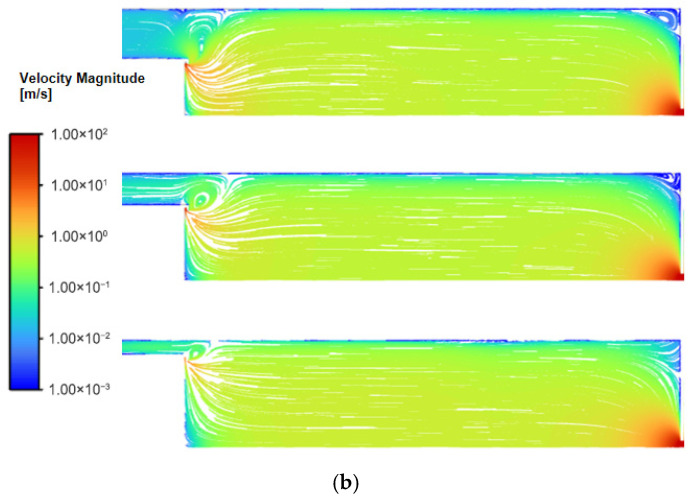
(**a**) Streamline diagram for different magnetron radii at 300 sccm. (**b**) Streamline diagram for different magnetron radii at 450 sccm.

As the inlet flow rate increases from 150 sccm to 300 sccm and 450 sccm, the flow field structure undergoes significant changes. Under higher flow conditions, gas is ejected at high speed from the annular inlet along the target surface, forming distinct closed streamlines in the region near the inlet and generating a vortex structure, as shown in Figure 7.

The size of the vortices is essentially confined to the gap between the magnetron and the chamber wall. Increasing the radius, which reduces the gap, can effectively minimize these vortices.

Definition of particle transmission rate:

(9) $$\eta =\frac{N_{out}}{N_{total}}$$

**Figure 8:**
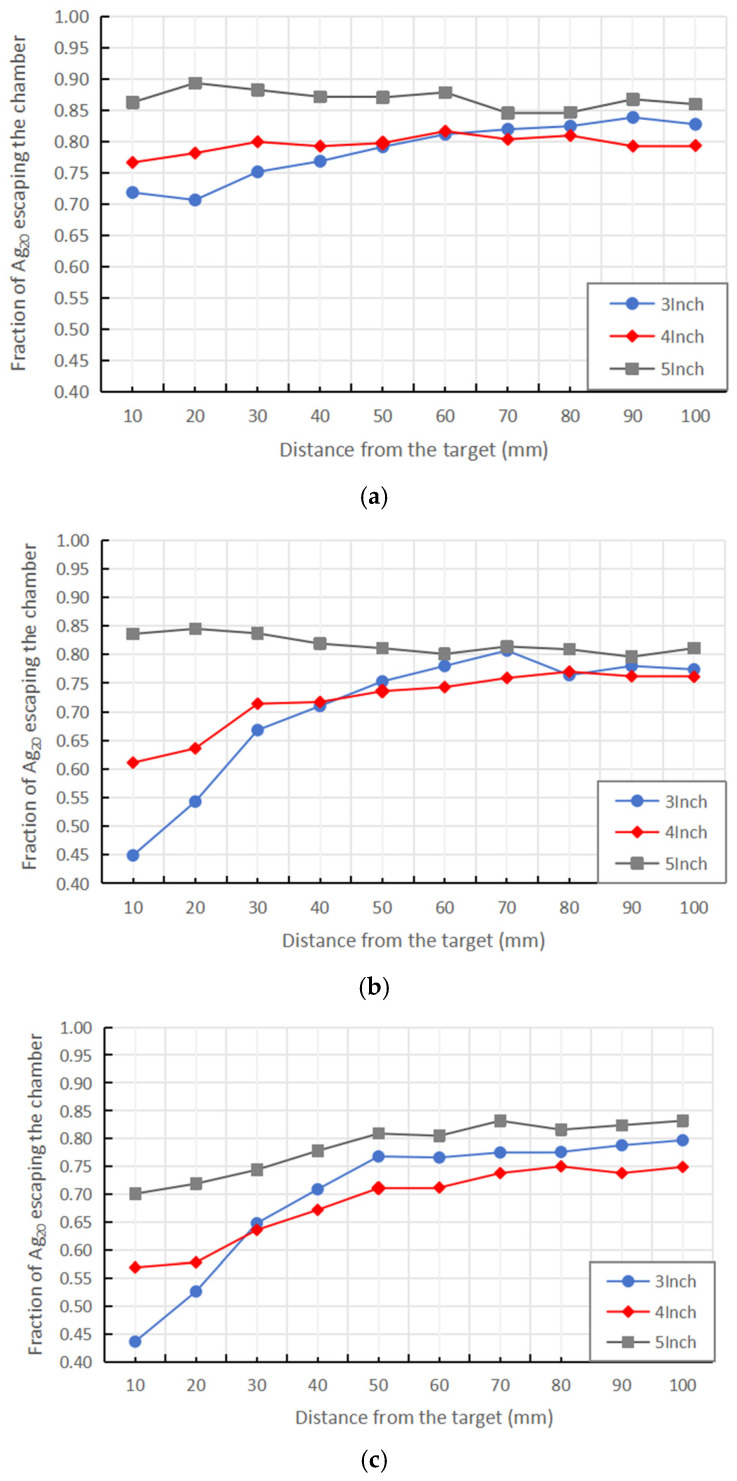
Particle transmission rate at different release distances. (**a**) 150 sccm. (**b**) 300 sccm. (**c**) 450 sccm.

The particle transmission rates at gas flow rates of 150, 300, and 450 sccm are shown in Figure 8.

Under the 150 sccm operating condition, the flow field structure corresponding to different magnetron radii is generally stable; no significant large-scale vortex structures were observed inside the chamber, and the flow is primarily axial transport. In the near-target region, 10–50 mm from the target surface, the particle transmission rate exhibits a clear upward trend as the magnetron radius increases.

As the magnetron radius increases, the annular gap between the outer wall of the magnetron and the chamber wall decreases. Under the same mass flow rate conditions, the average gas velocity in this region increases significantly, thereby enhancing the gas’s ability to transport particles axially.

Furthermore, as the magnetron radius increases, the inlet position gradually moves closer to the chamber wall. Local streamlines near the inlet tend to become more parallel to the wall.

Consistent with the flow-straightening mechanism described in Section 3.1, the particle transmission rate in the near-target region (10–50 mm from the target surface) increases monotonically with magnetron radius, as shown in Figure 8.

To quantify the extent of recirculation, the vortex area fraction $f_{vortex}$ is defined as the fraction of the flow cross-sectional area in the near-target region (0–100 mm downstream of the target surface) where vorticity magnitude exceeds 40 s^−1^:

(10) $$f_{vortex}=\frac{A_{\omega >40}}{A_{flow}}\times 100\%$$

This region is selected to isolate the recirculation structures most responsible for particle trapping, excluding the high-vorticity zones at the annular inlet jet and the outlet nozzle. The denominator $A_{flow}$ denotes the available flow cross-sectional area in the r–z plane for each magnetron configuration (32,300, 31,036, and 29,820 mm^2^ for the 3-inch, 4-inch, and 5-inch cases, respectively), accounting for the difference in annular gap width.

The resulting values are summarized in Table 3. The monotonic decrease in $f_{vortex}$ with increasing magnetron radius at all flow rates provides direct quantitative evidence that larger magnetron radii effectively suppress recirculation formation, corroborating the particle transmission trends reported in Section 3.2.

In the central region of the chamber, 60–100 mm from the target surface, the differences in particle transmission efficiency under different magnetron radius conditions decrease significantly, and the radial velocity distribution tends to become consistent.

This indicates that in the central region of the chamber, the flow field has fully developed into a relatively stable axial mainstream structure, with the flow pattern primarily controlled by the overall geometric structure, while the influence of the local inlet structure gradually weakens. Consequently, particle transport behavior in this region exhibits low sensitivity to the magnetron radius.

When the gas flow rate was increased to 300 sccm and 450 sccm, the flow field structure underwent significant changes. Under conditions of smaller magnetron radii (3 inches and 4 inches), local closed streamlines near the inlet formed distinct vortex structures. The vortex region causes particles entering this area to linger for an extended period, significantly increasing the probability of particles eventually colliding with the wall, which leads to a substantial decrease in particle transmission efficiency.

In contrast, under a 5-inch radius condition, only small-scale local vortex structures form near the inlet, and the overall flow field maintains a flow pattern dominated by axial transport. Since the vortex region is small, its interference with the main flow channel is limited, allowing particles to be efficiently transported axially to the outlet. Consequently, a high particle transmission rate is maintained throughout the entire study distance under high-flow conditions.

**Figure 9:**
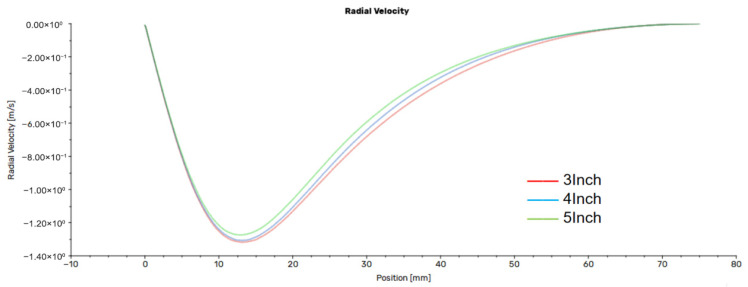
Radial velocity at 20 mm from the outlet under a flow rate of 450 sccm.

Defining the direction toward the center as negative radial velocity, the trends in radial velocity distribution are generally consistent across different flow rate conditions. Taking the cross-section at 20 mm from the outlet under a flow rate of 450 sccm as an example, as shown in Figure 9, the radial velocity reaches its maximum value at a distance of 10–20 mm from the center before decaying toward the axis and walls. Notably, the radial velocity correlates inversely with the magnetron radius. This phenomenon indicates that under smaller radii conditions, the gas emerges from the inlet with a higher initial radial velocity, causing the flow lines to bend significantly toward the center; whereas under larger radii conditions, the radial momentum at the inlet is reduced, resulting in smoother flow lines and consequently a reduced radial diffusion effect.

**Figure 10:**
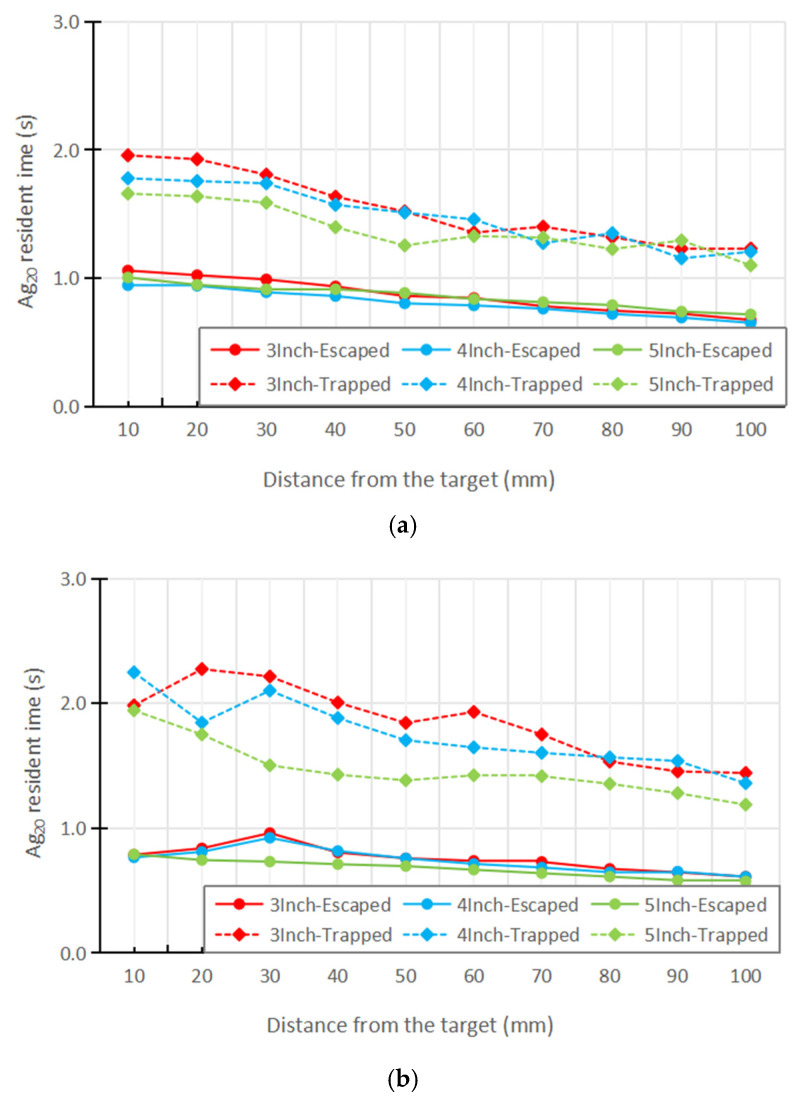

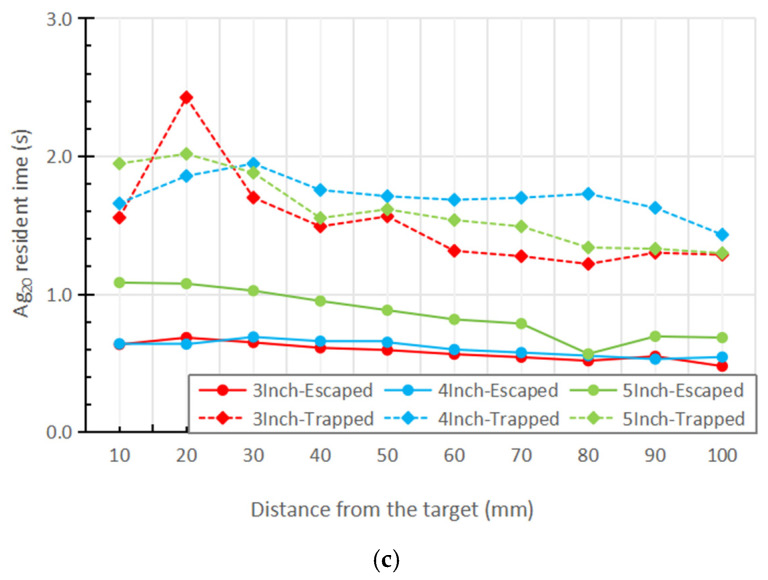
Average particle residence time. (**a**) 150 sccm. (**b**) 300 sccm. (**c**) 450 sccm.

Figure 10 presents the mean residence times of escaped and trapped Ag_20_. For escaped particles, residence times are approximately 0.6–1.0 s across all magnetron configurations and release distances, with limited variation between radii at 300 sccm. This uniformity reflects the dominant influence of Brownian motion on particles that successfully traverse the chamber: stochastic thermal forcing randomizes individual trajectories sufficiently that the mean escape time is governed primarily by the overall transit length rather than the local flow structure. At 450 sccm, the 5-inch configuration exhibits a notably elevated escaped residence time relative to the 3-inch and 4-inch cases, which is attributed to the extremely narrow annular gap (8.5 mm) generating high-velocity inlet jets that, in combination with Brownian perturbations, deflect a fraction of escaped particles away from the direct axial pathway, extending their in-chamber transit.

A more pronounced and physically informative contrast emerges in the trapped particle residence times. At 300 sccm, the 3-inch configuration exhibits a local maximum in mean trapped residence time at 20–30 mm from the target surface (~2.3 s), with the 4-inch configuration showing a similar but less pronounced elevation. This axial range corresponds precisely to the location of the vortex structures identified in the flow field analysis (Figure 7), confirming that particles released in this region are captured by the recirculation zone and undergo extended retention before eventually impinging on the chamber wall. At 450 sccm, the trapped residence time peak for the 3-inch configuration intensifies further (~2.4 s at 20 mm), consistent with the enlarged recirculation zone observed at higher flow rates.

In contrast, the absence of a residence time peak in the 5-inch case constitutes additional evidence that the recirculation zone is effectively suppressed at the larger magnetron radius.

Taken together, the trapped particle residence time distribution provides a spatially resolved signature of vortex activity that is robust to Brownian motion: while escape-time statistics are smoothed by thermal fluctuations, the vortex-induced delay in wall-impinging particles remains detectable as a localized elevation in the 20–30 mm region for configurations where recirculation is present.

**Figure 11:**
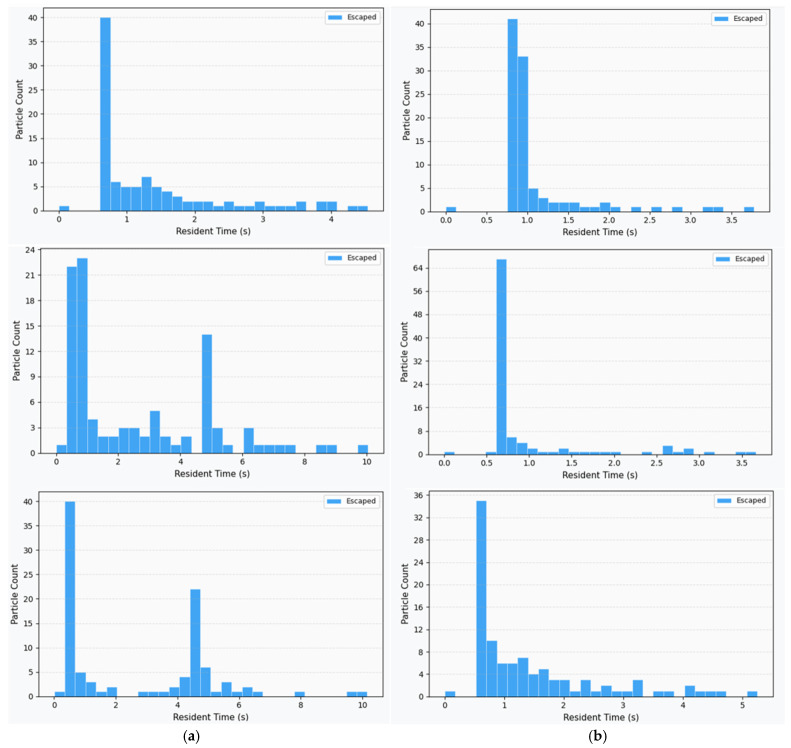
Residence time distribution (stack). (**a**) 3 Inch. (**b**) 5 Inch.

Figure 11 presents the residence time distributions of escaped particles for the 3-inch and 5-inch configurations at three flow rates. Only particles that successfully exited through the outlet are included; wall-trapped particles are excluded from this analysis.

At 150 sccm, both configurations exhibit a unimodal distribution concentrated near 1 s, indicating that at low flow rates, all escaped particles traverse the chamber via the direct axial pathway without significant interaction with recirculation structures.

At 300 and 450 sccm, the two configurations diverge markedly. The 5-inch case retains a unimodal distribution near 1 s at all flow rates, confirming that the suppressed recirculation zone allows escaped particles to follow a single, unobstructed transport pathway. In contrast, the 3-inch configuration develops a bimodal distribution with a primary peak near 1 s and a secondary peak near 4 s.

Critically, both populations in the 3-inch case represent particles that ultimately escaped the chamber. The primary peak corresponds to particles that traversed the main axial flow channel without entering the recirculation zone. The secondary peak, delayed by approximately 3 s, represents particles that were transiently captured by the vortex structures before eventually being re-entrained into the main flow and escaping through the outlet. This bimodality directly reveals the partial-trapping character of the recirculation zone: it does not exclusively deposit particles on the wall, but also imposes substantial transit delays on a fraction of the escaped population.

## 4. Conclusions

This study demonstrates that the magnetron radius plays a critical role in regulating the flow field structure, which in turn governs particle transport behavior and residence time within the condensation chamber under low-temperature, low-pressure conditions using CFD simulations. The main conclusions are as follows:

(1) Changes in the magnetron radius exert minimal impact on the global flow field topology of the chamber, but significantly modify the local velocity distribution, particularly the intensity of axial flow within the annular gap region.

(2) As the magnetron radius increases, the flow field gradually transitions from a flow pattern characterized by strong radial diffusion to one dominated by axial transport, thereby enhancing the orderliness of the flow.

(3) Particle transmission efficiency and growth opportunity increase significantly with magnetron radius, primarily due to enhanced axial flow and reduced vortex regions, which minimize particle stagnation and wall deposition.

(4) Under high-flow conditions, larger magnetron radii effectively mitigate the large-scale vortices observed at smaller radii and reduce particle loss thereby maintaining high throughput.

The realizable k–ε model provides a practical compromise between computational cost and accuracy for transitional flows; however, future work could employ more advanced closures such as the SST k–ω model or large-eddy simulation to better resolve near-wall and transitional flow features.

This work establishes a clear relationship between flow structure, residence time, and the conditions favorable for nanoparticle growth, offering valuable insights for the design and optimization of high-efficiency nanomaterial synthesis systems based on magnetron sputtering.

## Figures and Tables

**Table 1:** Mesh properties table.

Mesh	Cell Number	Maximum Skewness	Orthogonal Quality
Mesh 1	1.3 M	0.6827	0.3605
Mesh 2	0.8 M	0.7456	0.4179
Mesh 3	0.5 M	0.6801	0.4827

**Table 2:** Mesh independence verification table.

Mesh	Average Outlet Velocity (m/s)	Recirculation Area * (mm^2^)	Particle Transmission Rate (%)
Mesh 1	0.2272	3005	0.706
Mesh 2	0.2238	3001	0.711
Mesh 3	0.2589	2989	0.771

* Defined as the cross-sectional area where vorticity magnitude exceeds 40 s^−1^, evaluated within 0–100 mm of the target surface.

**Table 3:** Recirculation area fraction $f_{vortex}$(%) in the near-target region (0–100 mm from target surface, ω > 40 s^−1^) for different magnetron radii and flow rates.

Flow Rate (sccm)	3-Inch (%)	4-Inch (%)	5-Inch (%)
150	0. 929	0. 659	0. 597
300	2.014	1.522	1.058
450	3.02	2.210	1.011

## Data Availability

The original contributions presented in this study are included in the article. Further inquiries can be directed to the corresponding author(s).

## Abbreviations

The following abbreviations are used in this manuscript:

CFD	Computational Fluid Dynamics
DPM	Discrete Phase Model
CVD	Chemical Vapor Deposition
